# Chimeric antigens displaying GPR65 extracellular loops on a soluble scaffold enabled the discovery of antibodies, which recognized native receptor

**DOI:** 10.1080/21655979.2023.2299522

**Published:** 2024-01-07

**Authors:** Janine Barrett, Seppe Leysen, Cécile Galmiche, Hussein Al-Mossawi, Paul Bowness, Thomas E. Edwards, Alastair D.G. Lawson

**Affiliations:** a UK Research Department, UCB Pharma, Slough, UK; b Nuffield Department of Orthopaedics, Rheumatology and Musculoskeletal Sciences, University of Oxford, Oxford, UK; c US Research Department, UCB Pharma, Bainbridge Island, WA, USA

**Keywords:** GPCR, GPR65, phage display, chimeric protein antigens, monoclonal antibody generation

## Abstract

GPR65 is a proton-sensing G-protein coupled receptor associated with multiple immune-mediated inflammatory diseases, whose function is relatively poorly understood. With few reagents commercially available to probe the biology of receptor, generation of an anti-GPR65 monoclonal antibody was desired. Using soluble chimeric scaffolds, such as ApoE3, displaying the extracellular loops of GPR65, together with established phage display technology, native GPR65 loop-specific antibodies were identified. Phage-derived loop-binding antibodies recognized the wild-type native receptor to which they had not previously been exposed, generating confidence in the use of chimeric soluble proteins to act as efficient surrogates for membrane protein extracellular loop antigens. This technique provides promise for the rational design of chimeric antigens in facilitating the discovery of specific antibodies to GPCRs.

## Research Highlights

- This technique offers a viable approach for antibody discovery to difficult GPCRs.
- Structurally relevant, soluble chimeric scaffold proteins of GPR65 were generated.
- Chimeric antigens were used to identify GPR65-specific antibodies by phage display.

## Introduction

Ankylosing spondylitis (AS) is the prototypical seronegative spondyloarthritis, presenting with chronic inflammation primarily in the spine and sacroiliac joints, often in association with extra-articular features and comorbidities including inflammatory bowel disease and psoriasis [1,2]. While current therapeutic strategies for AS include the use of biologics to neutralize specific proinflammatory cytokines, many patients exhibit an inadequate clinical response to last-line therapies [3,4]. With significant unmet medical need, further research is required to fully understand the pathogenesis of this disease and elucidating the pathogenic role of genetically associated proteins will be key in developing effective therapeutics.

Genome wide association studies have identified GPR65 association with many immune-mediated diseases, including AS [5,6]; however, its pathogenic role remains unknown. Discovered in 1996, GPR65 is a proton-sensing G protein coupled receptor (GPCR) family member, which becomes optimally activated at pH 6.4–6.8, leading to G_s_ protein signaling and resulting in the accumulation of intracellular cAMP (cyclic adenosine monophosphate) [7,8]. GPR65 mRNA is predominantly expressed in the spleen, thymus, and peripheral blood leucocytes [9], therefore, the receptor is hypothesized to play an important role in the immune system.

GPCRs are highly conserved cell surface receptors which transduce extracellular signals into physiological effects and represent the largest family of proteins encoded by the human genome. Through their involvement in many key processes, their dysfunction contributes to many human diseases. Historically, there has been intense interest in the expansive GPCR family, and they still stand as the largest group of therapeutic targets, with most successful approaches targeting GPCRs with small molecules and peptides [10]. Despite decades of work, there are still only two approved GPCR targeted therapeutic antibodies, speaking to the technical challenges associated with identifying functional antibodies against GPCRs, and the need to advance extracellular loop display [11]. The creation of soluble antigens designed from the extracellular domains of calcitonin gene-related peptide linked to Fc domains enabled the discovery of erenumab, the first FDA approved antibody against a GPCR [12]. Alternatively, mogamulizumab was generated through the humanization of a chimeric mouse anti-CCR4 antibody, raised by immunizing a mouse with a peptide consisting of only 27 amino acids from human CCR4 [13,14].

Here, we use rationally designed chimeric protein scaffolds based on human apolipoprotein E3 (ApoE3) to present extracellular loops (ECL) of a GPCR as antigens in phage display, to discover GPR65-specific antibodies capable of recognizing native cell-expressed protein.

## Results

**Figure 1.**
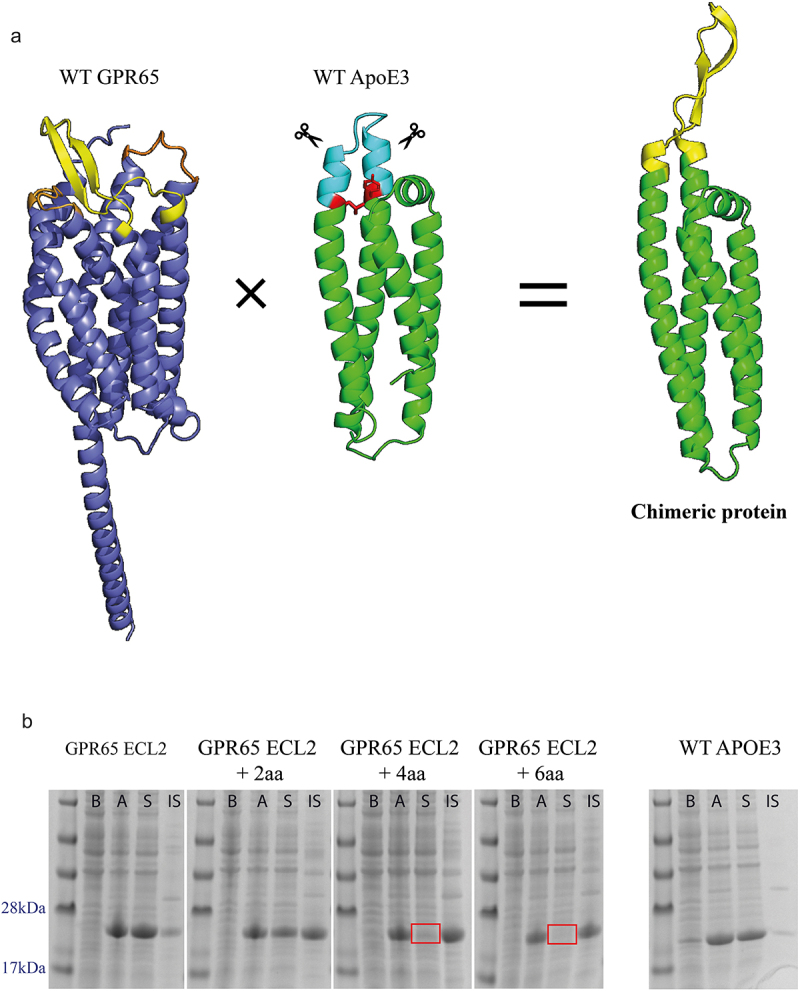
Expression of GPR65 chimeric constructs as soluble proteins. a) AlphaFold models representing human GPR65 (Uniprot entry Q8IYL9), truncated human apolipoprotein E (Uniprot entry P02649, PDB 1BZ4) and a representative example of the chimeric proteins, here displaying ECL2 of GPR65 (yellow) from the ApoE3 backbone (green). The transmembrane and ICL of GPR65 are in dark blue, with alternative GPR65 ECL in orange. Within the WT ApoE3 protein, the light blue region indicates the substituted residues, with red sites representing critical interactions between the two helices, which maintain structural integrity of the protein. b) SDS-PAGE gel of cell lysates before (b) and after (a) induction, and soluble (S) and insoluble (IS) fractions of expressed proteins for chimeric constructs with ECL2, representative for both ECL1 and ECL3. The + ‘x’aa represents how many amino acids on each side of the loop, as part of the helix, were also substituted with those from native GPR65. Correct bands are observed between 22 and 23kDa. Red squares represent a lack of soluble protein.

Work carried out in the vaccine field suggested that substituting structures of interest onto protein scaffolds, termed ‘epitope transplantation,’ can elicit an immune response to the antigen [15]. Within the seven-transmembrane structure of GPR65, the extracellular loops would likely exit from and enter back into alpha helices. Hence, three constructs were designed to utilize the 4 alpha-helix bundle structure of soluble ApoE3, with each of the preserved GPR65 ECL substituted for the wild type (WT) residues 105–109 (‘LGQST’) of ApoE3; in turn mimicking the membrane protein ECL leaving and entering a helix on each side (Figure 1a

).

Additional constructs were also designed to incorporate several amino acids of the helices that lead into and away from the loop from the GPR65 sequence, to see if a more representative reflection of the backbone would provide a closer structure of the loops within the native protein. Limits of these alterations along the two helices were calculated upon the identification of a seemingly critical interaction (Figure 1a, represented as red residues), required to maintain structural integrity of the protein. Finally, an additional His-Avi-TEV site was included within the construct design to aid purification of the protein, as well as biotinylation for downstream use.

Analysis of cell lysates, before and after induction, verified the expression of the chimeric proteins from bacteria. Next, the solubility of the chimeric proteins was assessed by separating the cell lysate (after induction) into a soluble and insoluble fraction through centrifugation. Majority of the proteins contained sequence changes that were well tolerated and found in the soluble fraction, whilst those where amino acids of the backbones were replaced with GPR65 residues (represented by + 2aa, +4aa and + 6aa), remained in the insoluble fraction (Figure 1b).

**Figure 2.**
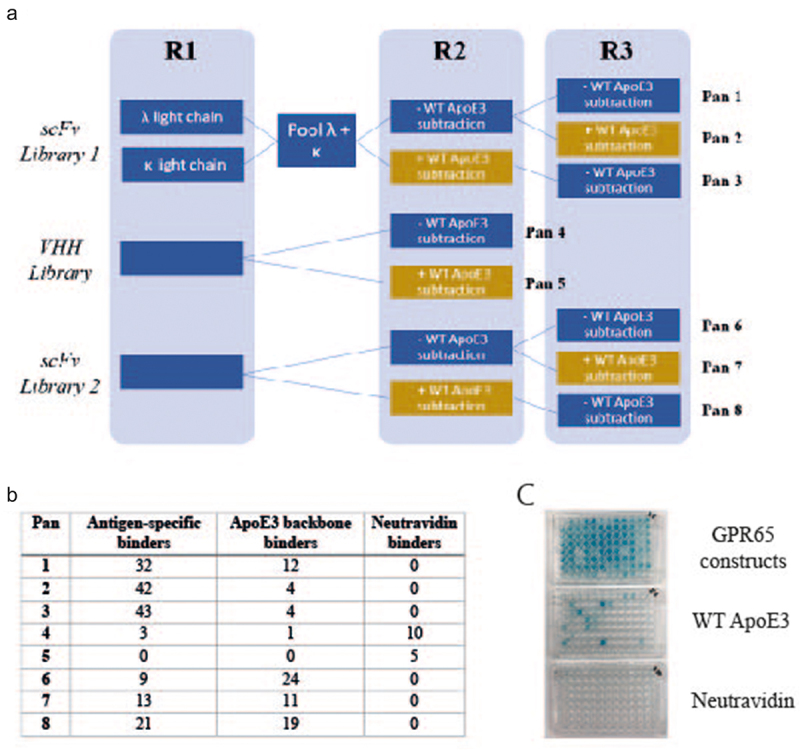
Generation of GPR65 antigen-specific monoclonal antibodies, using phage display. a) Schematic of phage display panning; three constructs representing each extracellular loop of GPR65 were pooled and acted as antigen throughout all stages of phage panning, for each of the three libraries screened (2 naïve scFv and naïve VHH). The antigen concentration remained the same throughout each round (1.5µM) and rounds 2 and 3 also included a subtraction step against WT ApoE3 (yellow boxes), to remove antibodies that bound the backbone. Eight pans were generated, each representing a different panning strategy. Phage antibody-containing supernatant was analyzed by ELISA against the pool of three chimeric constructs, WT ApoE3 and neutravidin. Those positive only on the construct plate were selected as antigen-specific hits, whilst those which also bound ApoE3 were deemed backbone binders. b) Binding results from each of the different panning conditions. c) Representative ELISA plates for pans 1 and 3 (half a plate each; pan 1 on the left-hand side, pan 3 on the right-hand side).

The chimeric proteins with direct loop substitutes went on to be used as antigens within a phage display campaign (Figure 2a) to pan 3 established UCB phage libraries. Two libraries of naïve human single chain variable fragments (scFv), each created independently from different donors, and a library of llama variable domain of heavy chain only antibodies (VHH) were included to increase diversity, aiming to expose the small (<30 amino acid) GPR65 ECL epitopes to advantageous antibody formats and diversity.

As peptides were acting as the antigen, the panning strategy was designed to increase efficiency by pooling all three constructs, each representing one of the GPR65 ECL, as well as maintaining a high concentration throughout the three panning rounds. Importantly, the epitopes of interest presented by the chimeric constructs were a small portion of the whole protein, therefore throughout the later panning rounds, subtraction pans were also incorporated. WT ApoE3 (Figure 2a, yellow boxes) was used to remove backbone binders and encourage only those recognizing the substituted loops of interest to become enriched.

Once panning was complete, monoclonal antibodies were rescued, and antibody-containing supernatant were screened by ELISA, identifying 163 antigen-specific binders (Figure 2b). Of the antibodies derived from the VHH library, the subtraction pan did not appear to enrich loop-specific binders, resulting in more from those not exposed to WT ApoE3. The scFv libraries led to a higher hit rate of antigen-specific antibodies; with enrichment of loop-specific antibodies through the inclusion of a subtraction step (at either rounds 2 or 3) and resulted in fewer ApoE3 backbone binders. The additional panning round for scFv libraries over the VHH library was due to library size differences (Figure 2b).

Upon phagemid DNA amplification by PCR, sequencing analysis identified 28 unique antibodies, 26 derived from the scFv libraries, and 2 from the VHH phage library. To screen for antibody activity, extracted VHH and scFv products were subjected to seamless cloning and reformatted into scFv-rabbit Fc or VHH-rabbit Fc formats; 20 phage antibodies were successfully cloned.

**Figure 3.**
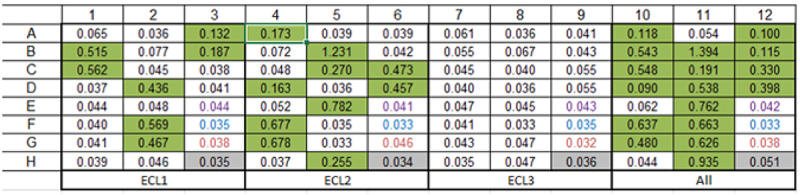
Phage derived antibodies specifically bound ECL1 and ECL2 GPR65 chimeric constructs. 20 cloned antibodies were expressed, and antibody-containing supernatant binding was tested by ELISA against plate-bound individual chimeric constructs or as a pool, to determine their specificity (numbers provided are optical density values). Layouts of antibodies tested were repeated across the plate, per each binding profile (ECL1, ECL2, ECL3 and a pool of all three in ‘All’). Binding is highlighted in green. The purple wells represent values from the secondary antibody control, the blue wells are blank controls, and the red values from mock transfected HEK cell supernatant, as an additional control.

The antibodies were transiently expressed, and binding specificity verified by ELISA. Of the 17 antibodies successfully expressed, loop-specific antibodies were confirmed to have been generated, with 7 recognizing the ECL1 construct, whilst 10 bound the ECL2 construct (Figure 3). Though expecting to see binding to ECL2 as the largest of the three loops, it was encouraging to also see binding to one of the smaller loops, ECL1.

**Figure 4.**
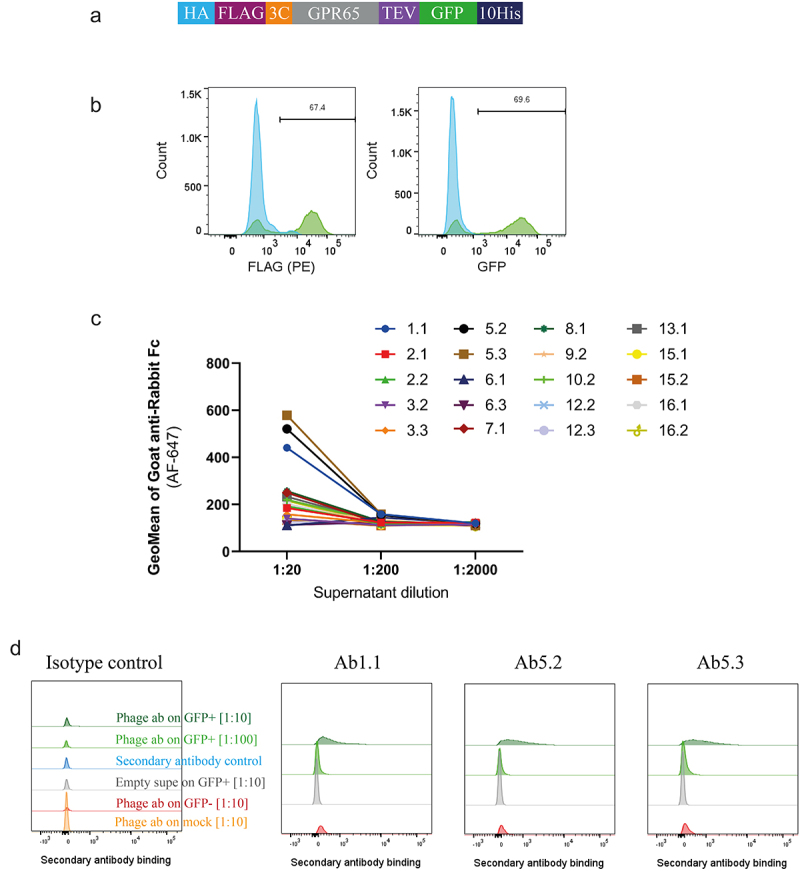
Phage-derived antibodies raised against chimeric constructs were able to recognise GPR65 expressed on HEK cells. a) GPR65 construct containing hemagglutinin (HA) signal sequence to encourage trafficking to the cell surface, FLAG tag to confirm surface expression, 3C and TEV protease sites to cleave off tags, His tag to aid with purification and GFP to aid with detection of gene expression. b) The construct was transiently transfected into HEK cells and incubated for 24 h. Binding was analyzed by flow cytometry, after initially gating on forward and side scatter, by FLAG expression, through a PE-conjugated anti-FLAG antibody, and detection of GFP. Wild type HEK cells are depicted in blue, whilst HEK cells transfected with human GPR65 are depicted in green. Percentage of GPR65 positive cells (green) is stated within the plots. Diluted phage-derived antibody-containing supernatant was incubated with HEK cells ±GPR65, and binding was detected using an anti-rabbit Fc. c) Initial HEK cell screen of all phage-derived antibodies, highlighting 3 titrating GPR65-specific binders. d) Flow cytometry plots of the three GPR65-specific binders where GFP^+^ cells represent GPR65 expressing cells (green), whilst GFP^−^ cells (reg and grey) represent the internal negative control of cells not successfully transfected with GPR65 DNA, while mock cells (orange) reflect WT HEK cells having undergone the transfection protocol, without the addition of any DNA.

A successful phage campaign delivered chimeric construct-specific antibodies, but it was important to understand whether these could recognize receptor expressed on a cell surface. Expression of both upstream FLAG and downstream GFP (Figure 4a, b) were used to imply surface expression of wild-type GPR65 upon being transiently transfected into HEK cells. In a primary binding screen on these cells, concentration-dependent binding to the receptor was observed for 3 of the phage-derived antibody-containing supernatants, compared to background (Figure 4c, d). The three binders specifically recognized ECL2 and represented the two distinct libraries panned; Ab1.1 coming from the VHH library (pan 4, without WT ApoE3 subtraction), and antibodies Ab5.2 and Ab5.3 derived from scFv library 1. Indeed, Ab5.2 and Ab5.3 came from the same campaign (pan 1, without WT ApoE3 subtraction) but being discovered independently increases confidence in the validity of the sequence. The consistent performance of these two clones throughout screening, further confirms the binding profile of this antibody and supported selection for further interrogation.

**Figure 5.**
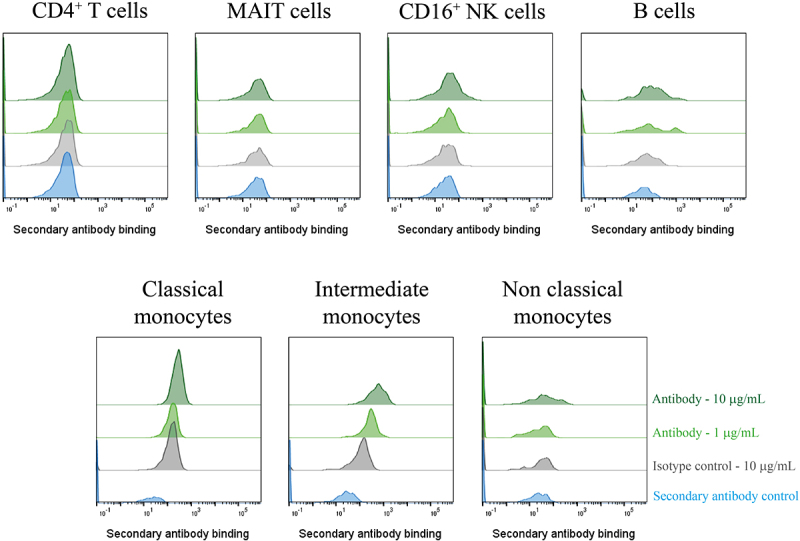
A GPR65-specific monoclonal antibody was successfully derived from phage display of a chimeric protein. PBMCs were isolated from a healthy control (*n* = 1), treated with Fc block for 10 min, then phage-derived antibody incubated with the cells for 1 h. DAPI-negative live cell subsets were identified according to their phenotype^1^ before secondary antibody (anti-rabbit IgG AF647) binding was assessed by flow cytometry. Phage-derived antibody binding is depicted in light or dark green, at 1µg/mL or 10 µg/mL respectively, with the secondary antibody control depicted in blue, and the isotype control reflected in grey. Ab1.1 is the example shown.

To remove any unwanted signals from artifacts that may have been in the supernatant, the three GPR65-specific antibodies were purified by Protein A tips (Phynexus) and were confirmed to maintain binding to GPR65 expressed on HEK cells. Binding of these antibodies was then assessed against primary immune cell subsets, to verify binding to native receptor *ex-vivo*. At this stage, only Ab1.1 presented population right shifts of binding, which appeared to titrate out, on CD16^+^ NK cells, B cells and monocytes, suggesting recognition of the receptor expressed on the surface of these cells; whilst not binding to T cells (Figure 5).

## Discussion

Here, chimeric antigens have been shown to be a viable approach to the discovery of antibodies to difficult membrane protein targets, through the display of GPR65 extracellular loops on a soluble scaffold.

Antibodies being used as tool reagents is not a new concept, with many specifically developed to understand target biology and function, or to aid with structural studies. There is currently no gold standard for antibody generation to any given target, with a broad variety of strategies used in campaigns, to leverage upon each of their advantages and disadvantages. Antibodies derived from antigen-specific B cells upon immunization of a host have already undergone affinity maturation, have a high specificity and provide low immunogenicity. Whilst display technologies offer the screening of large libraries covering millions of V(D)J combinations, compared to the circulating average 10^7^ in humans, ultimately improving the chance of identifying rare antibodies [16,17].

Due to known difficulties of immunizing hosts with small epitope, membrane expressed proteins, identifying a GPR65 antibody through phage display was favored. For optimal success, the antigen being presented needs to be as similar to its native conformation as possible. Without reliable recombinant protein, screening against whole cells expressing the target is an alternative solution, particularly for GPCRs as native conformation is presented. However, this approach also has its own limitations. Low abundance of membrane protein expressed on cells, coupled with a high background of other proteins displayed on the surface of a cell do not create the best environment for identifying target-specific binders, particularly here where small extracellular domains were the site of interest. Moreover, phage particles are notoriously sticky and have been known to be internalized by cell membrane receptors [16], shrinking the library being screened. Thus, a novel strategy for antigen presentation is desired.

Mindful of the approach of Correia and colleagues [15], chimeric constructs were designed and wild type GPR65 extracellular loops were successfully displayed on the alpha-helix of soluble ApoE3, when substituted at the specific joining site between the loop and alpha-helix. The lack of solubility of those constructs where amino acids of the backbones were replaced with GPR65 residues, suggested an alteration to the structure of the scaffold. While it is possible that the additional residues could contribute directly to the insolubility (e.g., FNAVML and INLNLF, represented the longest addition of amino acids at both the ends of the loop substitution), it seems more likely that structural integrity of the chimeras was compromised, possibly causing the proteins to misfold and aggregate into inclusion bodies and highlighting the importance of maintaining the integrity of the alpha helices.

We note that Koksal *et al*, report an antibody scaffold mimetic (ASM) in which the N terminus and all three extracellular loops of CXCR4 were presented in the complementarity-determining region of an antibody [18]. It is perhaps somewhat fortuitous that the juxtaposition of the loops was sufficiently similar in the ASM for useful antibodies to be obtained. The method we describe here enables antibodies to individual loops to be obtained, and of course these individual loops can be presented in separate constructs and used for immunizations and panning in parallel. We also believe that splicing onto alpha helical scaffolds in ApoE3 is more relevant and structurally representative for GPCR antigens than being supported on the beta sheet of an antibody scaffold. Although we have no experience of double substitution into adjacent loops in ApoE3, we would not necessarily advise following such an approach when using broadly for alternative GPCRs. Instead, one would recommend grafting each loop individually into separate ApoE3 frameworks, and possibly mixing as immunogens.

Within the human libraries screened, the multiple rounds of panning allowed for subtraction through enrichment, minimizing attrition at later stages. Overall, the phage display campaign was a success, with 17 antibodies discovered which bound the chimeric antigens, possessing specificity for the GPR65 substituted loops. It is of note, that pooling of antigens did not appear to bias antibodies dominant for one construct. Monoclonal antibodies recognizing both ECL1 and ECL2 were identified, whilst no antibodies were recovered for ECL3.

Without the ability to screen for binding to GPR65 recombinant protein, three phage-derived antibodies were shown to specifically recognize GPR65 expressed on HEK cells, with binding comparisons between the WT GPR65 receptor, alternative membrane proteins and mock-transfected cell lines; thus, validating the technique for using scaffold proteins to display antigen and elicit a response to facilitate the identification of native antigen-specific binders. However, for these antibodies to be useful for interrogating GPR65 biology, it was important they were able to bind endogenous receptor expressed on primary human cells.

We saw convincing binding of the VHH Ab1.1 to B cells, monocytes and NK cells, but not to CD4^+^ T cells or mucosal-associated invariant T (MAIT) cells. To date, published data reporting GPR65 expression has been obtained through mRNA analysis, with MAIT cells, CD16^+^ NK cells and non-classical monocytes as the highest expressors [19]. Through the inclusion of an Fc block step, the Fc portion of an antibody should have been prevented from interacting with FcγRIII (CD16) expressed on NK cells and non-classical monocytes.

The lack of binding at either concentration to MAIT cells was unexpected, although it is not unusual to observe discrepancies between mRNA and protein expression of a given target due to posttranscriptional processing and regulation [20]; therefore, transcripts are not sufficient to predict protein levels [21]. However, if mRNA expression is high, there is a correlation for protein to be produced, even if not to the same high level. At the time of writing, there are no reports of GPR65 protein being expressed on MAIT cells. It is possible that the presentation and conformation of ECL2 in MAIT cells differs from other cell types, with potential occlusion of the loop from close binding of unreported proteins in a complex or differential glycosylation. In this context, it is of interest that Ab1.1 the VHH with the smallest footprint, showed binding to GPR65 on primary cells, while the scFv antibodies featuring larger paratopes were unable to bind. Further work is required to improve the antibody properties of Ab1.1, to be able to confidently use it as a tool reagent for probing GPR65 biology, in health and disease.

In addition, it is important to note that there is a potential N-linked site (NWT) in substituted ECL1 and another (NFT) in substituted ECL2 of the chimeric constructs, but it is not clear if these are used. Mammalian expression could readily be used for the expression of the chimeras, but variable and unrepresentative glycosylation could lead to antibodies with less than universal application in studies of native GPR65. Glycosylation can lead to occlusion of antibody-binding sites, but in this case antibodies, which bound to HEK expressed and primary cell GPR65 were discovered from panning against *E.coli*-produced constructs.

In summary, although applied so far to GPR65, the work presented here provides evidence to support a scaffold-based approach for displaying looped epitopes, resulting in the identification of native protein loop-specific antibodies. We see no reason why the method cannot be of general applicability with accurate, structurally enabled chimeras able to be readily constructed using the acceptor framework presented here. Not only does this technique provide promise for future display technology campaigns, but also within immunization strategies; using host species scaffolds to present targets with traditionally unstable structures to elicit a targeted immune response. With enhancements of research on basic structure, as well as developments of novel approaches for antibody discovery against difficult structures, the generation of functional antibodies against difficult to target GPCRs is closer than ever before.

## Methods

### Chimeric protein expression and purification

Genes encoding ApoE3 chimeric constructs were synthesized by ATUM, and cloned into BamHI and NcoI restriction sites of a pET14b vector. These constructs were co-transformed with a pRSFDuet-1 vector encoding Biotin ligase BirA, into NiCo21(DE3) *E. coli* cells (New England Biolabs). Expression and purification of the constructs were performed, as described by the Swanstrom lab [22], with addition of 100 µM biotin to the growth medium at the time of induction with IPTG. Protein concentration was determined on a Nanodrop spectrophotometer, using absorption at 280 nm (Thermo Scientific). To determine the solubility of each protein upon expression in *E. coli*, SDS-PAGE was performed. Briefly, samples taken from the cultures, before and after induction, were lysed using BugBuster MasterMix. After taking a sample from the lysate of the induced culture sample, it was further separated in a soluble and insoluble fraction by centrifugation. NuPAGE™ LDS Sample Buffer 4× (Invitrogen) containing dithiothreitol (Invitrogen), was added to cell lysates, soluble protein fraction and insoluble protein pellets to a final 1X concentration, before being run along a NuPAGE™ 4–12% Bis-Tris Protein Gel (Invitrogen). Instant blue™ (Expedeon) was used to reveal protein bands.

### Phage panning in solution

Three UCB established phage libraries were used; two naïve human scFv phage libraries and a naïve VHH phage library.

In the first round of panning, ApoE3 GPR65 loop constructs (1.5 µM) were pooled and incubated with each library, previously blocked in PBS containing 3% milk. Constructs and associated phage were captured from solution using Sera-Mag™ SpeedBeads Neutravidin-coated (GE Life Sciences). Beads were washed by magnetic capture 5 times with PBS + 0.1% TWEEN (VWR), and bound phage were eluted by 100 µg/mL trypsin (Sigma) in Tris-buffered saline buffer. Mid-log growing TG1 cells were infected with these phage particles and used for output titrations and overnight amplification. Rescued phage from round one was split in two and incubated directly with either the same pool of antigen, or 1.5 µM WT ApoE3 construct, to remove binders to the ApoE3 scaffold. Complexes were captured using Dynabeads™ *M*-280 Streptavidin (Invitrogen), to remove neutravidin binders, followed by selection on chimeric ApoE3 GPR65 proteins. Enriched phage from round two of both human libraries were subject to a final round, and complexes were captured with neutravidin beads. As the VHH library was smaller than the human libraries, only two rounds of panning were carried out. Phage rescue was performed from polyclonal *E. coli* populations between rounds or from individual *E. coli* colonies after the final round.

### Phage rescue

Phage rescue was performed using a modification of the standard protocol [23]. Cultures were seeded at a starting density of 0.1 OD_600_ in 2×YT medium with 2% glucose and 100 μg/mL carbenicillin and grown at 37°C with shaking at 220 rpm until the OD_600_ reached between 0.4 and 0.8. The culture was then infected with M13KO7 at a multiplicity of infection of 10 and incubated at 37°C for 1 h, without shaking. The culture was then centrifuged at 4000 rpm for 10 min. The pellets were resuspended in 100 mL 2×YT containing 100 μg/mL carbenicillin and 50 μg/mL kanamycin. These cultures were grown for 18 h at 30°C, shaking at 240 rpm. Next, the cultures were centrifuged at 8000 rpm for 15 min and phage-containing supernatants were used for ELISA screening or precipitated for further selection rounds through incubation with 7 mL PEG sodium chloride for 1 h, on ice.

### Phage antibody characterisation and optimisation

Monoclonal phage antibodies were screened for binding to the antigen they were raised against, WT ApoE3 and neutravidin, by ELISA. Briefly, biotinylated antigen (chimeric constructs and WT ApoE3) or neutravidin was coated onto ELISA plates at 1 µg/mL. Plates were blocked with PBS with 3% milk (Sigma), before 100 µL of pre-blocked phage supernatant was applied to the plate. Following a wash step (composition of ELISA wash is detailed in Supplementary Table S1), anti-M13 HRP (Sino Biological) was added at 1:15000 dilution. Plates were developed with TMB, before being read at 630 nm, on Synergy 2 (BioTek).

Phagemid DNA was sequenced (Macrogen), before unique scFv/VHH regions were extracted, amplified, and reformatted into a rabbit Fc fusion vector, by seamless cloning (GeneArt™ Seamless Cloning and Assembly Enzyme Mix).

The unique antibodies were synthesized and cloned into custom vectors by TWIST Bioscience.

### DNA constructs and preparations

A human GPR65 DNA construct was designed (Uniprot entry Q8IYL9) along with a N-terminal FLAG tag, a C-terminal 10-histidine tag and labeled with GFP. Custom synthesis and cloning into a mammalian expression vector were performed by ATUM (California, USA). Plasmid DNA was amplified through Qiagen Plasmid Plus Giga kits and quantified.

### Antibody and target transient expression

HEK293 cells were cultured in EXPI293 expression media (Life Technologies), and cell concentration and viability were determined using trypan blue (Gibco, Life Technologies). Cells were routinely cultured at 37°C, in 8% CO_2_, in vented Erlenmeyer flasks (Corning, Surrey, UK), shaking at 180 rpm and sub-cultured every 3–4 days, at a seeding density of 0.5 × 10^6^ cells/mL.

Cells were transfected with DNA-lipid complexes comprising DNA and Expifectamine293 (Life Technologies) at a 1 µg DNA:1 or 3 × 10^6^/mL cell ratio for target and antibodies, respectively, and prepared according to manufacturer’s protocol. Transfected cells were incubated for 24 h, or up to 7 days, respectively, prior to use.

### Isolation of peripheral blood mononuclear cells (PBMCs)

Venous blood samples from anonymous healthy donors based at UCB Celltech, Slough, UK were taken directly into heparin-containing tubes. Blood samples were taken with informed consent under UCB Celltech HTA License number 12,504, as approved by the Human Tissue Authority. All donors gave written informed consent in accordance with the Declaration of Helsinki.

Sample was diluted 1:1 with PBS and mononuclear cells were separated from whole blood using Leucosep™ tubes. These were centrifuged at 800×g for 15 min with no brake, and the PBMC-containing interface was collected using a Pasteur pipette. Cells were washed twice in PBS containing 1 mM EDTA (here on in referred to as PBS+EDTA) for 10 min at 200×g, then resuspended in R10. Cells were counted after staining with trypan blue using a hemocytometer under a light microscope.

### Flow cytometry

Monoclonal antibodies used here are listed in Supplementary Table S2. Data acquisition was obtained using a BD Bioscience Canto II. Fluorescence was compensated using single color compensations Ultracomp beads (eBioscience) and data were analyzed using FlowJo v9 or newer (BD Life Sciences).

### Surface staining

Up to 2 × 10^5^ cells/well were stained in FACS buffer (composition of buffer is detailed in Supplementary Table S1), in a total volume of 50 µL per well. Cells were incubated for up to 1 hour at 4°C in the dark with a panel of fluorochrome conjugated antibodies. Samples were then washed twice in FACS buffer and resuspended in up to 200 µL for acquisition (with DAPI (Biolegend)).

Where primary and secondary antibodies were used, cells were first stained with the primary antibody as described above, washed twice in FACS buffer, and stained with secondary antibody for a further 30 min. Instances where Fc block (BD Biosciences) was used, 2.5 µg/1×10^6^ cells was added for 10 min prior to surface antibody incubation.

## Abbreviations

ApoE3: Apolipoprotein E 3
AS: Ankylosing spondylitis
CCR4: C-C chemokine receptor 4
DNA: Deoxyribonucleic acid
ECL: Extracellular loop
ELISA: Enzyme-linked immunosorbent assay
FDA: Food and Drug Administration
GFP: Green fluorescence protein
GPCR: G-protein coupled receptor
HEK: Human embryonic kidney 293 cells
ICL: Intracellular loop
MAIT: Mucosal-associated invariant T cell
mRNA: Messenger ribonucleic acid
NK cell: Natural killer cell
PBMC: Peripheral blood mononuclear cells
PCR: Polymerase chain reaction
scFv: Single chain variable fragment
VHH: Single variable domain on a heavy chain
WT: Wild type

## Supplementary Material

Original Image Figure 1B Part 1.jpg

Original Image Figure 1B Part 2.jpg

## Data Availability

The data that support this study are available from the corresponding author, JB, upon reasonable request.

## Note

1. CD4+ T cells = CD3+, CD4+MAIT cells = CD3+, CD8+, CD161+, Vα7.2+CD16+ NK cells = CD3-, CD56+, CD16+B cells = CD3-, CD19+Classical monocytes = CD3-, CD14+, CD16-Intermediate monocytes = CD3-, CD14+, CD16+Non-classical monocytes = CD3-, CD14-, CD16+

## Additional Information

This work was supported by UCB Pharma; no further funding was received.
