# Supplementary figures and images for: Chimeric antigens displaying GPR65 extracellular loops on a soluble scaffold enabled the discovery of antibodies, which recognized native receptor
Source: Bioengineered. 2024 Jan 7;15(1):2299522. doi: 10.1080/21655979.2023.2299522 (PMC10773626; doi:10.1080/21655979.2023.2299522)

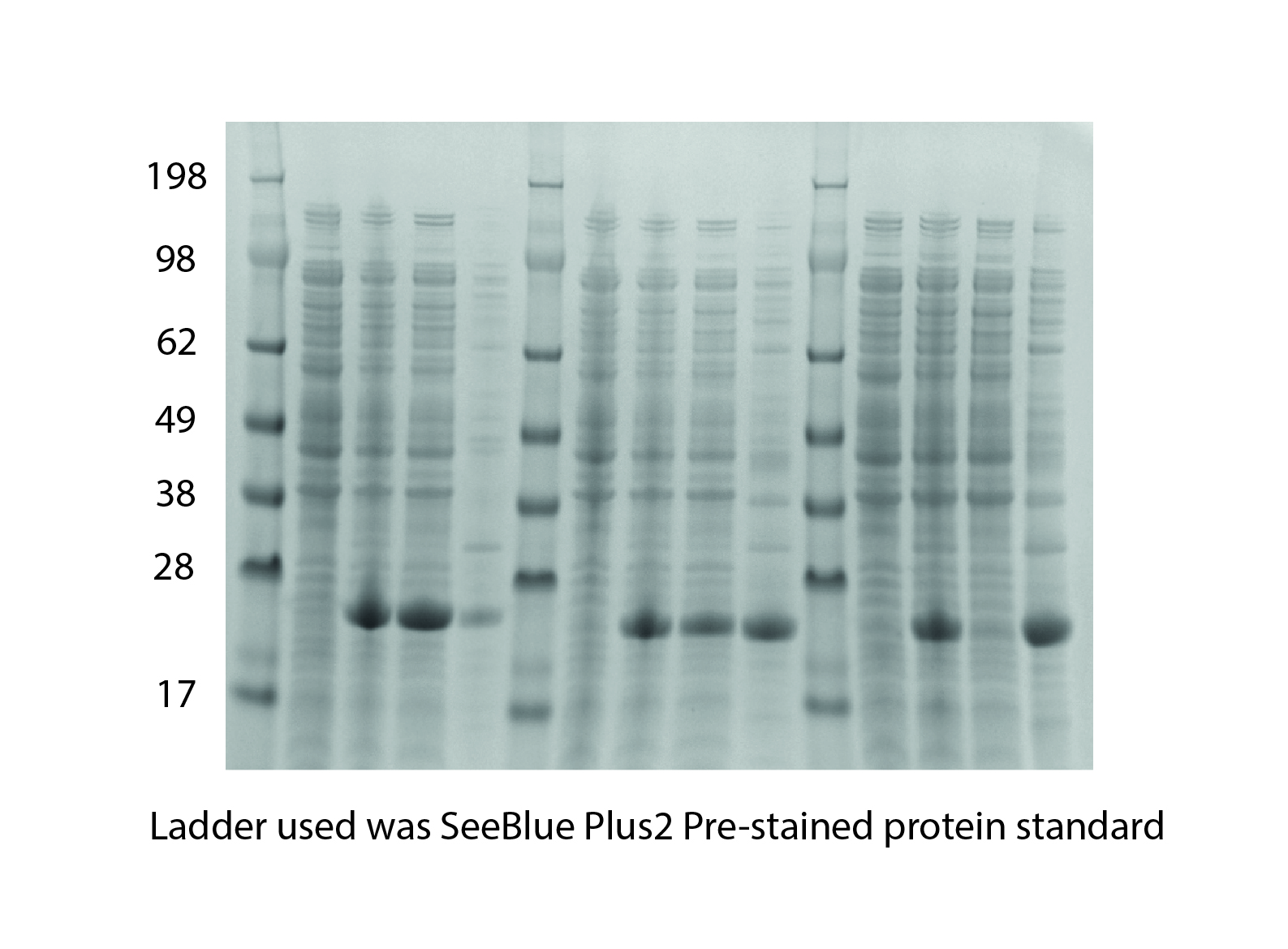

Supplement: Original Image Figure 1B Part 1.jpg [file KBIE_A_2299522_SM0401.jpg]

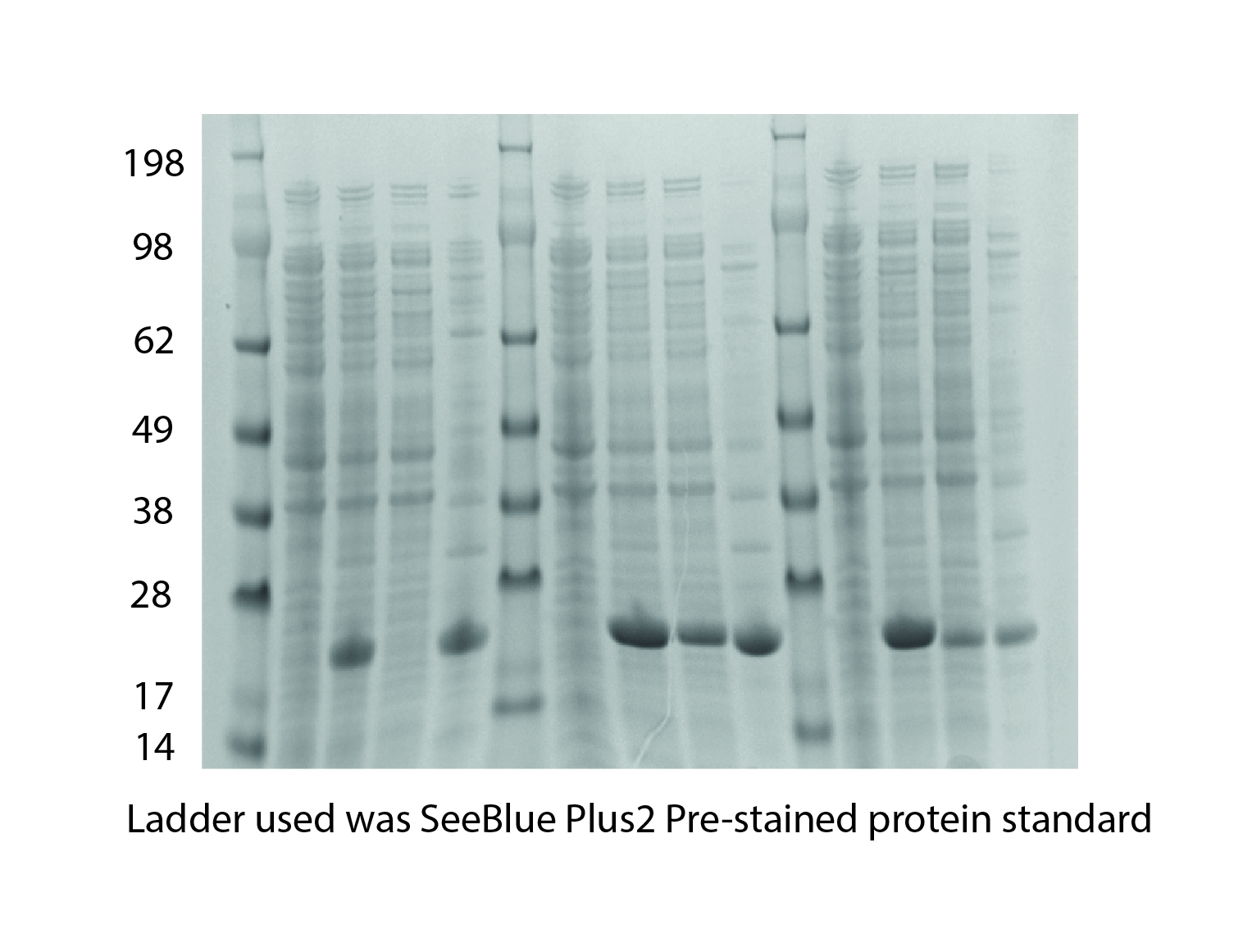

Supplement: Original Image Figure 1B Part 2.jpg [file KBIE_A_2299522_SM0400.jpg]
